# An uncommon spontaneous right distal tubal pregnancy post bilateral laparoscopic sterilization: A case report

**DOI:** 10.1097/MD.0000000000014193

**Published:** 2019-01-25

**Authors:** Ching-Min Lin, Yu-Lun Ku, Yu-Tzu Cheng, Ngo Yeh Giin, Yu-Che Ou, Meng-Chih Lee, Chung-Yuan Lee

**Affiliations:** a Department of Obstetrics and Gynecology, Chia-Yi Chang Gung Memorial Hospital; b Department of Nursing, Chang Gung University of Science and Technology, Chia-Yi Campus, Chia-Yi; c Institute of Medicine, Chung Shan Medical University, Taichung; d School of Post-Baccalaureate Medicine, Kaohsiung Medical University, Kaohsiung, Taiwan.

**Keywords:** ectopic pregnancy, fallopian tube pregnancy, laparoscopic tubal sterilization, sterilization

## Abstract

**Rationale:**

Tubal sterilization as a contraception method has a high success rate; however, it also carries a low risk of incidental pregnancy. A majority of these pregnancies are ectopic. In this study, we report a rare case of spontaneous right distal tubal pregnancy after bilateral laparoscopic tubal sterilization.

**Patient concerns:**

A 36-year-old woman who had undergone bilateral laparoscopic tubal sterilization presented with abdominal pain and a positive test for pregnancy.

**Diagnosis:**

Ectopic pregnancy was suspected based on absence of gestational sac in the uterine cavity on ultrasound and elevated beta-human chorionic gonadotropin (β-hCG) level.

**Intervention:**

Since the patient had unstable vitals, emergency laparoscopic surgery was performed, which revealed a right distal fallopian tube pregnancy. We performed a complete bilateral residual tubal stump excision.

**Outcomes:**

The patient recovered well after surgery, with a reduction in β-hCG level, and was discharged after 3 days.

**Lessons:**

To ensure complete sterilization, the gap at the excised end needs to be adequately widened and enhanced with electro-destruction to prevent formation of a fistula.

## 1 Introduction

Tubal sterilization has a high rate of success in preventing pregnancy, and it is, therefore, a common form of contraception for women. Pregnancy after tubal sterilization is uncommon and is relatively rare. In the United States of America, the 10-year cumulative probability of pregnancy after sterilization was reported to be 18.5 per 1000 procedures.^[1]^ When pregnancy does occur in such cases, it is generally ectopic. The exact pathogenesis of ectopic pregnancy after tubal sterilization remains unknown. A fallopian tube pregnancy at the distal end, after both fallopian tubes have been ligated, is very rare. In this report, we present a case of remnant distal tubal pregnancy, post-laparoscopic bilateral tubal sterilization. We also compare this case with other previously reported cases of ectopic pregnancy after sterilization.

## 2 Case report

A 36-year-old woman with an obstetric history of gravida 6, para 3 (all normal spontaneous deliveries), and 2 induced abortions, presented to our emergency department with an acute and persistent dull pain in the right lower quadrant of her abdomen, that had started a few hours before her arrival. This was accompanied by additional signs and symptoms including rebound pain, cold sweating, and nausea without vomiting. There were no relieving or aggravating factors. Her last menstrual cycle was 7 weeks and 4 days ago, and she had no history of pelvic inflammatory disease.

**Figure 1:**
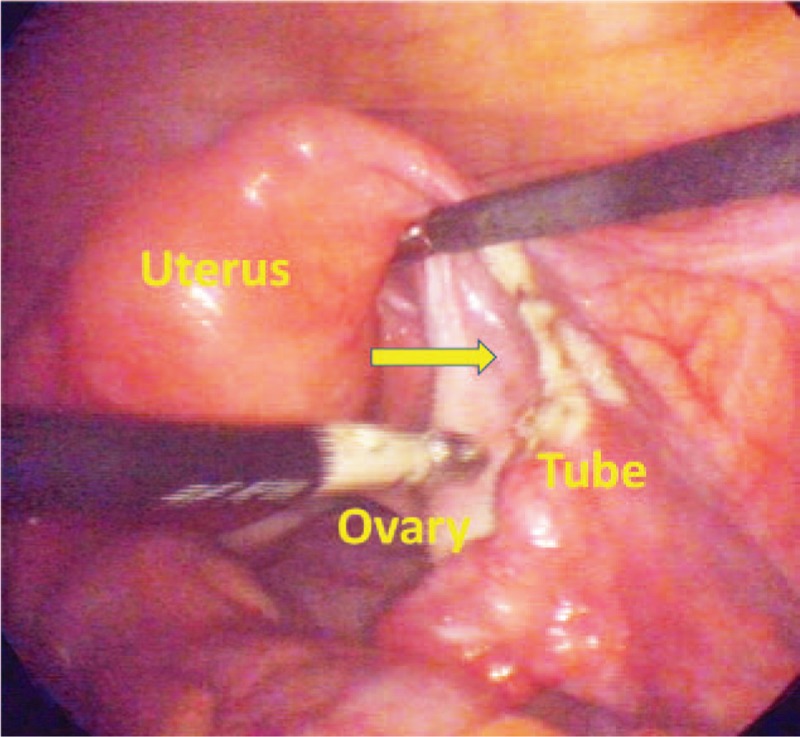
Result of laparoscopic tubal sterilization operation. An estimated 2 cm gap between the proximal and distal blunt ends. The arrow denotes the site of tubal ligation.

Her past medical history included human papilloma virus infection, administration of 3 doses of Gardasil vaccination (7 years ago), intrauterine device insertion (4 years ago), diagnosis of cervical intraepithelial neoplasia 3, and a cervical conization procedure (3.5 years ago), and laparoscopic tubal sterilization (1-year-4-months ago). Figure 1 presents the result of her laparoscopic tubal sterilization surgery. According to our image records, there was an estimated 2 cm gap between the proximal and distal blunt ends.

Initial physical examination showed stable vitals. Abdominal palpation revealed generalized abdominal stiffness and tenderness. Urine pregnancy test was positive. Transvaginal ultrasound showed no evidence of intrauterine pregnancy but revealed a right adnexal mass and presence of minimal ascites. Laboratory data revealed serum human chorionic gonadotropin (β-hCG) level of 15795 million IU/mL, white blood cells count of 12300/μL, and hemoglobin level of 11.9 g/dL.

Based on the clinical signs, imaging, and high level of serum β-hCG, an ectopic pregnancy was highly suspected, and she was admitted for a next day surgical intervention. However, 14 hours after admission to the gynecology ward, she experienced vomiting, sudden syncope, and hypotension (79/42 mm Hg). Laboratory test revealed that her hemoglobin had dropped to 8.9 g/dL. Based on a suspicion that the ruptured ectopic pregnancy might have caused hypovolemic shock, an emergency laparoscopic operation was performed.

**Figure 2:**
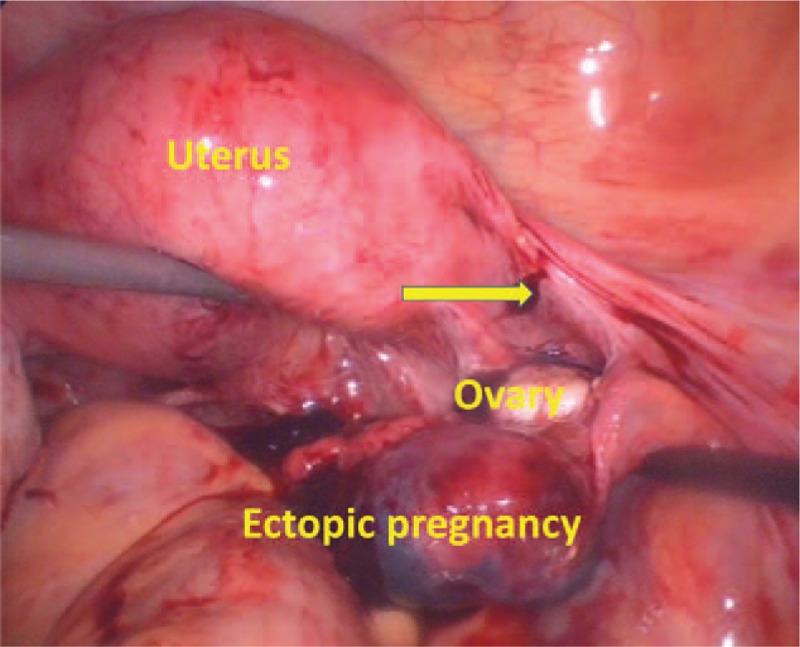
An emergency laparoscopic surgery found a 3 cm × 4 cm bulging mass at the right distal end of the remnant stump in the ampulla region. The arrow denotes the site of the previous tubal ligation.

Laparoscopy revealed a 3 cm × 4 cm bulging mass at the right distal end of the remnant stump in the ampulla region, and 1000 mL of internal bleeding. The gap at the site of previous right tubal sterilization was estimated to be 3.5 cm (Fig. 2). Both ovaries were normal in shape and size. No endometriotic foci or endometrioma were found. To mitigate the risk of a second ectopic pregnancy and in accordance with the patient's lack of desire for continued fertility, we performed a complete bilateral residual tubal stump excision. Histopathological assessment confirmed the diagnosis of right distal tubal stump ectopic pregnancy. One day post-operation, the B-hCG level reduced to 3968 million IU/mL. The patient recovered well after surgery and was discharged after 3 days.

## 3 Discussion

All methods of contraception have a risk of failure. Ectopic pregnancy after tubal sterilization, though extremely rare, is not impossible. The cause of ectopic pregnancy after tubal sterilization remains unclear. We performed a literature review of case reports from PubMed and MEDLINE from 1946 to present, using the keywords “ectopic pregnancy” and “tubal sterilization”, while including only cases of ectopic pregnancy post-sterilization and excluded cases with previous pelvic inflammatory disease or re-anastomosis operations. The results are summarized in Table 1.^[2–5]^ Sites of ectopic pregnancy include the ovary, omentum, broad ligament, and distal end of the remnant fallopian tube.

**Table 1:**
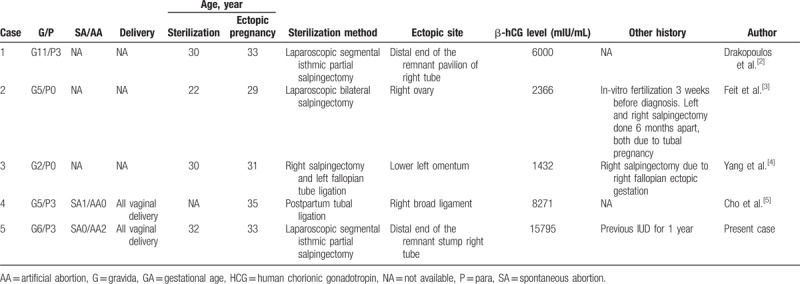
Characteristics of patients with ectopic pregnancy after sterilization.

According to the findings from the US Collaborative Review of Sterilization, the sterilization method, and age at sterilization influence the 10-year cumulative probability of ectopic pregnancy.^[1]^ This review concluded that women who underwent elective non-postpartum partial salpingectomy had a much greater risk of ectopic pregnancy than those who underwent postpartum partial salpingectomy. This is consistent with the findings of a review of literature. Of the 5 reported cases, 4 cases including ours underwent interval partial salpingectomy while only 1 underwent postpartum partial salpingectomy.

As reported by Shah et al,^[6]^ the possible mechanisms for ectopic pregnancy after tubal sterilization include tubal ligation recanalization, tubo-peritoneal fistula, or space formation in the altered tubal lumen. In the case presented here, we infer that a micro fistula had formed and therefore sperm penetration was possible through the blunt end. As the micro fistula was not large enough to allow the fertilized ovum to pass, distal segment implantation had occurred, resulting in ectopic pregnancy. Therefore, in order to have complete tubal sterilization to reduce the incidence of ectopic pregnancy, we suggest that the gap needs to be adequately wide and surgeons must enhance electro destruction of the excised edge to prevent fistula formation. Total salpingectomy should also be considered to prevent ectopic pregnancy, as this is an additional benefit, which reduces the risk for ovarian cancer. According to Kirsten et al,^[7]^ bilateral tubal ligation and total salpingectomy can reduce ovarian cancer risk by 13% to 41% and 42% to 78%, respectively. As removal of the fallopian tubes is also an effective prophylactic measure against ovarian cancer, it should be considered as an option for women who wish to undergo sterilization.

## 4 Conclusion

Ectopic pregnancy after tubal sterilization is extremely rare. In our case, we found that a micro fistula formation had resulted in sperm penetration through the blunt end. Based on this finding and previous literature, we suggest that in order to ensure complete tubal sterilization, the gap at the excised end needs to be adequate and surgeons must enhance electro destruction of the excised edge to prevent fistula formation.

## Acknowledgments

The authors would like to thank Chang Gung Medical Foundation Institutional Review Board for approving this case report for publication.

## Author contributions

**Conceptualization:** Chung-Yuan Lee.

**Data curation:** Ching-Min Lin, Yu-Lun Ku, Yu-Tzu Cheng, Ngo Yeh Giin, Chung-Yuan Lee.

**Formal analysis:** Ching-Min Lin, Chung-Yuan Lee.

**Investigation:** Ching-Min Lin, Chung-Yuan Lee.

**Methodology:** Chung-Yuan Lee.

**Project administration:** Ching-Min Lin, Yu-Lun Ku, Yu-Tzu Cheng, Ngo Yeh Giin.

**Resources:** Chung-Yuan Lee.

**Software:** Ching-Min Lin, Chung-Yuan Lee.

**Supervision:** Yu-Che Ou, Meng-Chih Lee, Chung-Yuan Lee.

**Validation:** Yu-Che Ou, Meng-Chih Lee, Chung-Yuan Lee.

**Visualization:** Chung-Yuan Lee.

**Writing – original draft:** Ching-Min Lin, Chung-Yuan Lee.

**Writing – review & editing:** Ching-Min Lin, Chung-Yuan Lee.

Chung-Yuan Lee orcid: 0000-0001-8838-0666.
